# Artificial Protein Cage Delivers Active Protein Cargos to the Cell Interior

**DOI:** 10.1021/acs.biomac.1c00630

**Published:** 2021-09-09

**Authors:** Antonina Naskalska, Kinga Borzęcka-Solarz, Jan Różycki, Izabela Stupka, Michał Bochenek, Elżbieta Pyza, Jonathan G. Heddle

**Affiliations:** † Malopolska Centre of Biotechnology, Jagiellonian University, 30-387 Krakow, Poland; ‡ Postgraduate School of Molecular Medicine, Medical University of Warsaw, Żwirki i Wigury 61, 02-091 Warsaw, Poland; § Institute of Zoology and Biomedical Research, Jagiellonian University, 30-387 Krakow, Poland

## Abstract

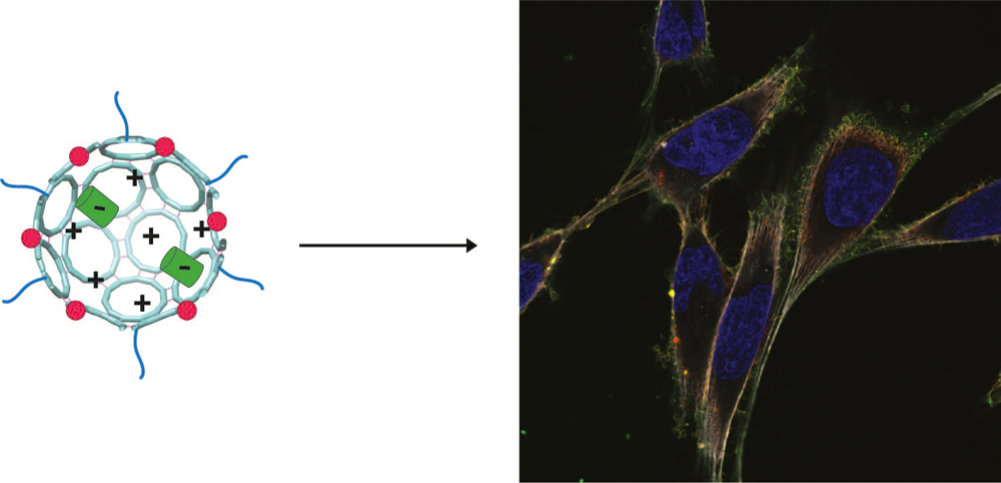

Artificial protein cages have potential as programmable, protective carriers of fragile macromolecules to cells. While natural cages and VLPs have been extensively exploited, the use of artificial cages to deliver active proteins to cells has not yet been shown. TRAP-cage is an artificial protein cage with an unusual geometry and extremely high stability, which can be triggered to break apart in the presence of cellular reducing agents. Here, we demonstrate that TRAP-cage can be filled with a protein cargo and decorated with a cell-penetrating peptide, allowing it to enter cells. Tracking of both the TRAP-cage and the cargo shows that the protein of interest can be successfully delivered intracellularly in the active form. These results provide a valuable proof of concept for the further development of TRAP-cage as a delivery platform.

## Introduction

Transport of molecular cargos to cells is desirable for a range of applications including delivery of drugs, genetic material, or enzymes. A number of nanoparticles have been employed to achieve this including liposomes,^1^ virus-like particles,^2^ nonviral protein cages,^3^ DNA origami cages,^4,5^ and inorganic nanoparticles.^6^ Protein cages are a promising approach as demonstrated by viruses in nature which are able to deliver genetic material to cells, often with high effectiveness and specificity. Adenoviruses, for example, are highly efficient and bind quite specifically to the CAR receptor.^7^

Artificial protein cages are constructed by proteins which do not naturally form cage structures and in which interactions between constituent proteins are modified to promote their assembly. The advantage of using such an approach is that the resulting cages can be given properties and capabilities that may not be available or feasible in naturally occurring forms. This includes triggerable assembly,^8,9^ which allows substituent proteins to be expressed as relatively small individual subunits with/without cargos, thus circumventing possible production problems associated with large complex formation in the cell. Artificial cages also allow other features such as geometries not seen in natural cages,^8^ which widens the possible library of building blocks, and replacement of enhancement of protein–protein interactions with other interactions, leading to control of disassembly.^8,10^ To date, a number of artificial protein cages have been produced including tandem fusions of proteins with two- and threefold rotational symmetries able to form a 12-subunit tetrahedral cage,^11−13^ a nanocube structure of 24 subunits with an octahedral symmetry,^14^ a 60-subunit icosahedral cage structure that self-assembles from trimeric protein building blocks,^15^ and co-assembling, two-component 120-subunit icosahedral protein complexes comparable to those of small viral capsids^9^ and designed peptides able to form networks that close to form cages.^16^ Several examples exist where artificial protein cages have been filled with various cargos including siRNA,^17,18^ mRNA,^18,19^ and fluorescent dyes.^10,20^ However, only a handful of cases have demonstrated delivery of cargos to cells by artificial cages.^17,20,21^ To the best of our knowledge, delivery of protein cargos to cells mediated by artificial protein cages (as opposed to natural cages) has not previously been demonstrated. In this work, we show for the first time that an artificial protein cage is capable of delivering a functional protein cargo to the cell interior.

**Figure 1:**
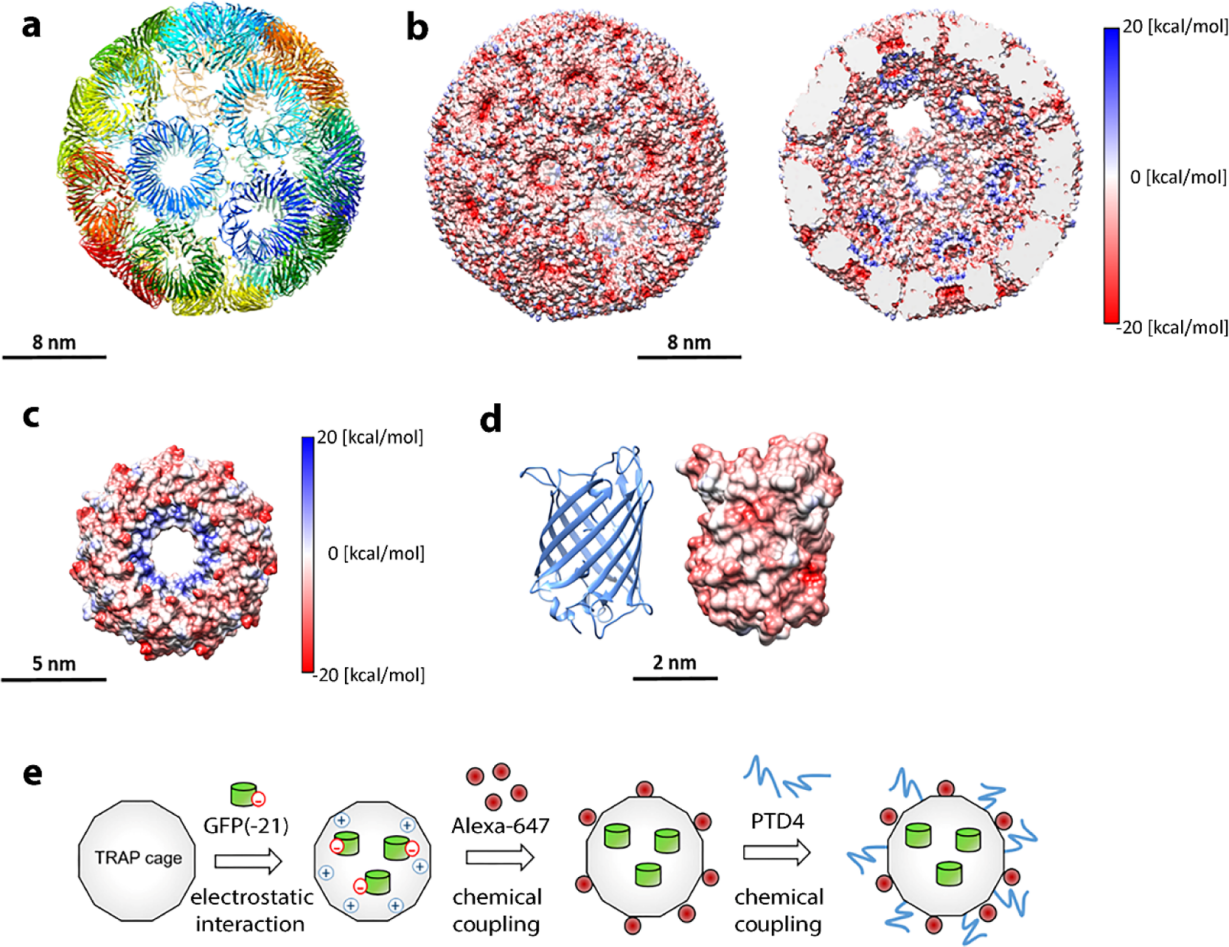
TRAP-cage protein. (a) Structure of TRAP-cage (PDB:6RVV) with each TRAP-ring shown in a different color. Gold atoms are shown as yellow spheres. (b) Surface representation of the TRAP-cage exterior (left) and interior (right) colored by charge distribution. (c) Surface view of a single TRAP-ring with the face that points into the interior cavity shown, colored according to the charge. (d) Negatively supercharged GFP(-21) shown in cartoon representation (left) and surface view colored according to charge (right). (e) Scheme of TRAP-cage encapsulation with GFP(-21) and external modifications with Alexa-647 dye and PTD4 peptide.

We previously produced an artificial protein cage using a building block consisting of the naturally occurring ring-shaped protein, TRAP (trp RNA-binding attenuation protein), referred to as TRAP-cage (Figure 1a).^8,22,23^ In nature, TRAP is involved in control of tryptophan synthesis and has been well characterized structurally and biochemically.^24−27^ It has also been used as a versatile building block in bionanoscience.^28−30^ TRAP-cage consists of 24 TRAP rings, forming an approximately 22 nm-diameter, 2.2 MDa hollow sphere with a lumen roughly 16 nm in diameter. Each TRAP ring in the cage is bound to five TRAP ring neighbors, and the structure contains six square holes approximately 4 nm in diameter. Unusually, compared to other natural and most artificial cages, the ring subunits in the cage are held together not by a network of protein–protein interactions. Instead, single gold(I) ions bridge opposing thiols of the cysteine residues between rings in proteins where the naturally occurring lysine at position 35 is replaced with cysteine. The cysteines of 10 of the 11 monomers of each ring in the cage are bridged in this way with those of the eleventh remaining unbridged and available to react, for example, with maleimide-labeled dyes. As the constituent TRAP ring is made from 11 monomers, it approximates a hendecagon, which would not be expected to assemble to form a regular-faced convex polyhedron. However, by assembling into an approximation of an Archimedean solid (a snub cube) and allowing for small amounts of deviation/flexibility, an “almost regular” TRAP-cage can form.^8^

TRAP-cage is extremely stable and able to survive temperatures of 95 °C for at least 3 h and high levels of denaturing agents such as 7 M urea.^8^ Despite this high stability, TRAP-cage breaks apart readily in the presence of low concentrations of reducing agents including the cellular reducing agent glutathione.^8^ This feature raises the prospect that the TRAP-cage may have utility as a system for delivering cargos to cells, as it can be expected to retain its structure, protecting the cargo until entering cells where intracellular reducing agents will result in disassembly and subsequent cargo release.

Here, we show that TRAP-cage can be deliberately filled with a protein cargo, and we use a negatively supercharged variant of green fluorescent protein, GFP(-21), as an exemplar molecule. Furthermore, we show that TRAP-cage can be used to deliver such cargos to the interiors of human cells. This cell penetration is itself controllable, as it only occurs if the surface of TRAP-cage is modified, for example, by cell-penetrating peptide. The results are a first step toward development of TRAP-cage as a potentially useful tool for delivering medically relevant cargos to cells and more generally demonstrate the potential for artificial protein cage systems as therapeutic agents.

## Materials and Methods

### Production and Purification of TRAP-Cage Filled with GFP(-21)

TRAP-cage (TRAP monomer sequence: MYTNSDFVVIKALEDGVNVIGLTRGADTRFHH-SEKLDKGEVLIAQFTEHTSAIKVRGKAYIQTRHGVIESEGKK) production and purification were performed as described previously.^8^ Supercharged (-21) His-tagged GFP protein (MGHHHHHHGSACELMVSKGEELFTGVVPILVELDGDVNGHEFSVRGEGEGDATEGELTLKFICTTGKLPVPWPTLVTTLTYGVQCFSRYPDHMKQHDFFKSAMPEGYVQERTISFKDDGTYKTRAEVKFEGDTLVNRIELKGIDFKEDGNILGHKLEYNFNSHDVYITADKQENGIKAEFEIRHNVEDGSVQLADHYQQNTPIGDGPVLLPDDHYLSTESALSKDPNEKRDHMVLLEFVTAAGITHGMDELYK^31^) was expressed from pET28a encoding the GFP gene and produced in BL21(DE3) cells. The protein was purified using Ni-NTA. Briefly, cells were lysed by sonication at 4 °C in 50 mM Tris-HCl, pH 7.9, 150 mM NaCl, 5 mM MgCl_2_, and 5 mM CaCl_2_, in the presence of protease inhibitors (Thermo Fisher Scientific), and lysates were centrifuged at 20 000 *g* for 0.5 h at 4 °C. The supernatant was incubated with agarose beads coupled with Ni^2+^-bound nitrilotriacetic acid (His-Pur Ni-NTA, Thermo Fisher Scientific) pre-equilibrated in 50 mM Tris, pH 7.9, 150 mM NaCl, and 20 mM imidazole (buffer A). After three washes of the resin (with buffer A), the protein was eluted with 50 mM Tris, pH 7.9, 150 mM NaCl, and 300 mM imidazole (buffer B). Fractions containing His-tagged GFP(-21) were pooled and subjected to size exclusion chromatography on a HiLoad 26/600 Superdex 200 pg column (GE Healthcare) in 50 mM Tris-HCl, pH 7.9, and 150 mM NaCl at room temperature. Protein concentrations were measured using a Nanodrop spectrophotometer using a wavelength of 280 nm.

GFP encapsulation was conducted by mixing equal volumes of 100 μM negatively supercharged (-21) His-tagged GFP with 1 μM pre-formed TRAP-cage incubated overnight in 50 mM Tris and 150 mM NaCl (pH 7.9). Purification of TRAP loaded with GFP(-21) was carried out by size exclusion chromatography using a Superose 6 Increase 10/300 column (GE Healthcare) in 50 mM HEPES, pH 7.5, and 150 mM NaCl. Fractions containing TRAP-cage were collected and analyzed by native PAGE using 3–12% native Bis-Tris gels (Life Technologies) followed by fluorescence detection using a Chemidoc detector (BioRad) with excitation at 488 nm.

### Ni-NTA “Pull Down”

Samples of purified TRAP-cage filled with His-tagged GFP(-21) protein were divided into two portions and incubated under reducing (1 mM TCEP) or nonreducing (no TCEP) conditions. Next, samples were passed through a Ni-NTA resin (Thermo Fisher Scientific) under gravitational flow, in which 100 μL of each sample was introduced onto 50 μL of the resin equilibrated with buffer A. Three samples were collected: (i) flow through, (ii) wash with buffer A, and (iii) elution with buffer B. Samples were analyzed by native PAGE, followed by fluorescence detection (excitation at 488 nm, Chemidoc, BioRad) and western blot. For the SDS-PAGE and western blot, samples collected from the Ni-NTA pull-down assay were denatured by addition of TCEP (final concentration 0.1 mM) and boiling for 15 min followed by separation via Tris/Glycine gel electrophoresis. Gels were then subjected to electrotransfer (2 h, 90 V) in 25 mM Tris, 192 mM glycine, and 20% methanol buffer onto an activated PVDF membrane. The membrane was blocked with 5% skimmed milk in Tris-buffered saline supplemented with 0.05% Tween 20 (TBS-T), followed by 1.5 h of incubation with the mouse monoclonal anti-GFP antibody (1:2500; St. John’s Laboratories, UK) and anti-mouse (1:5000, Thermo Fisher Scientific) secondary antibody conjugated with horse radish peroxidase. The signal was developed using a Pierce ECL western blotting substrate (Thermo Fisher Scientific) and visualized in a BioRad Chemidoc detector.

### TRAP-Cage Labeling with Alexa-647 and Decoration with Cell-Penetrating Peptide

Alexa Fluor-647 C2 maleimide fluorescent dye (Alexa-647, Thermo Fisher Scientific) and cell-penetrating PTD4 peptide were conjugated to the TRAP-cage filled with GFP(-21) via crosslinking reactions with cysteines and lysines present in the TRAP protein.

To achieve fluorescent labeling, TRAP-cage carrying GFP(-21) (300 μL, 16 nM) was mixed with a Alexa-647 C2 maleimide dye (50 μL, 1 μM); the reaction was conducted in 50 mM HEPES with 150 mM NaCl, pH 7.5, for 2.5 h at room temperature with continuous stirring at 450 rpm. The optimal interaction ratio of maleimide-conjugated Alexa-647 to TRAP-cage was assessed by titration (Figure S3a). Briefly, aliquots of TRAP-cage loaded with GFP(-21) (11.36 nM) were mixed with maleimide-conjugated Alexa-647 at concentrations ranging from 0.1 to 100 μM. Samples were then separated by native gel electrophoresis and visualized by fluorescence detection in a Chemidoc system, with excitation at 647 nm. Reactions where no free Alexa-647 is present in the sample and no GFP(-21) interference with the Alexa-647 signal is observed were considered to possess optimal decoration conditions and used in further experiments. The yield of TRAP-cage labeling with Alexa-647 was quantified using fluorescence detection of the labeled TRAP protein.

Additionally, to rule out the possibility of direct GFP(-21) labeling by Alexa-647, TRAP-cage loaded with GFP(-21) with and without Alexa-647 labeling was subjected to denaturing gel separation and western blotting followed by detection with the anti-GFP antibody.

For the cell-penetrating peptide decoration, PTD4 peptide (50 μL, 0.5 mM) was mixed with 1-ethyl-3-(-3-dimethylaminopropyl) carbodiimide hydrochloride (EDC, 10 μL, 83 mM) and *N*-hydroxysuccinimide (NHS, 10 μl, 435 mM), all reagents dissolved in ddH_2_O. Subsequently, an excess of activated PTD4 peptides was added to TRAP-cage filled with GFP(-21) and labeled with Alexa-647 and incubated for 2.5 h at room temperature, with continuous stirring at 450 rpm. The reaction was stopped by addition of 5 μL of 200 mM Tris-HCl, pH 7.5. The conjugation efficiency was verified by native PAGE, fluorescent gel imaging, and HPLC analysis.

### Electron Microscopy

TRAP-cage filled with GFP(-21), TRAP-cage filled with GFP(-21) and labeled with Alexa-647, and TRAP-cage filled with GFP(-21) and fully decorated were imaged using transmission electron microscopy (TEM). Samples were typically diluted to a final protein concentration of 0.025 mg/mL and centrifuged at 10,000*g* for 5 min at room temperature, and the supernatant was applied onto hydrophilized carbon-coated copper grids (STEM Co.) Samples were then negatively stained with 3% phosphotungstic acid, pH 8, and visualized using a JEOL JEM-2100 instrument operated at 80 kV.

### Flow Cytometry

For TRAP-cage internalization experiments, MCF-7 and HeLa cells were seeded into 12-well plates (VWR) in 800 μL of DMEM medium with 10% FBS at a density of 2.5 × 10^5^ per well and cultured for further 16 h prior to the experiments. Cells were then incubated with 50 μg (6 nM) of TRAP-cage filled with the cargo, labeled with Alexa-647 only, or decorated with Alexa-647 and PTD4 peptide in 50 mM HEPES with 150 mM NaCl, pH 7.5, supplemented with 10% FBS for 15 min, 2, and 4 h at 37 °C, 5% CO_2_. After the incubation, cells were washed three times for 5 min with phosphate-buffered saline (PBS) (EURx), harvested with trypsin (1 mg/mL), and centrifuged at 150*g* for 5 min. Subsequently, cells were washed thrice in PBS by centrifugation (150*g* for 3 min) and re-suspended in 0.5 mL of PBS. Cells were run in a Navios flow cytometer (Beckman Coulter), and the fluorescence of 12,000 cells was collected for each sample. Untreated cells and cells treated with TRAP-cage filled with cargo and labeled with Alexa-647 only were used as negative controls. Obtained data for three independent experiments were analyzed with Kaluza software (Beckman Coulter). The percentage of Alexa-647/GFP(-21) positive cells and median fluorescence intensity was determined for each sample.

### Laser Scanning Confocal Microscopy

For fluorescent laser scanning confocal microscopy observations, cells were grown on 15 mm glass cover slips plated into 12-well plates (2.5 × 10^5^ per well in 800 μL of DMEM medium with 10% FBS) and further stimulated with 50 μg (6 nM) of TRAP-cage filled with the cargo, labeled with Alexa-647 only, or decorated with Alexa-647 and PTD4 peptide in 50 mM HEPES with 150 mM NaCl, pH 7.5, supplemented with 10% FBS for 4 h at 37 °C and 5% CO_2_. Next, cells were washed with PBS (three times for 5 min), fixed with 4% paraformaldehyde solution (15 min, at room temperature), and permeabilized with 0.5% Triton-X100 in PBS (7 min, at room temperature). Actin filaments were stained with phalloidin conjugated to Alexa-568 in PBS (1:300, Thermo Fisher Scientific, 1.5 h, at room temperature). Cover slips were then mounted on slides using Prolong Diamond medium with DAPI (Thermo Fisher Scientific). Fluorescent images were acquired under an Axio Observer.Z1 inverted microscope (Carl Zeiss, Jena, Germany), equipped with the LSM 880 confocal module with a 63× oil immersion objective. Images were processed using ImageJ 1.47v (National Institute of Health).

## Results and Discussion

### Filling of TRAP-Cage

**Figure 2:**
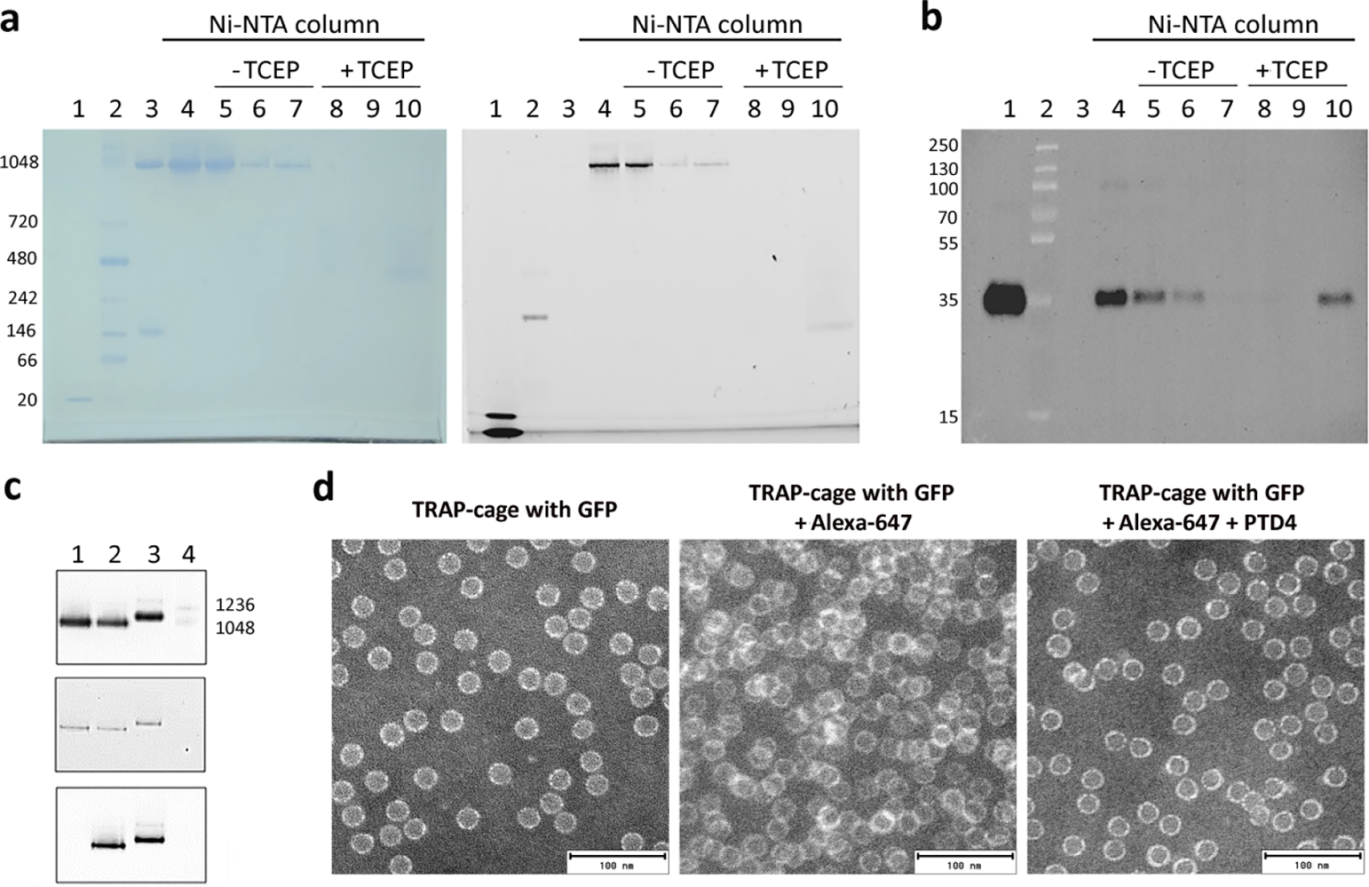
Filling and decoration of TRAP-cage. (a) Native PAGE gels showing purified TRAP-cage incubated with His-tagged GFP(-21) after passing through a Ni-NTA column in the absence (−TCEP) or presence (+TCEP) of TCEP. Lane 1: GFP(-21) positive control; 2: molecular weight marker for native PAGE; 3: empty TRAP-cage; 4: input [TRAP-cage with GFP(-21)]; 5 and 8: flow through; 6 and 9: wash; and 7 and 10: elution. Collected fractions were stained for protein (left) or analyzed by fluorescence detection (right, exct. 488 nm). The GFP signal visible in lane 10 of the right-side gel likely reflects GFP still bound to a TRAP ring. (b) Western blot of the gel in (a): collected fractions were subjected to SDS-PAGE followed by western blot with anti-GFP detection. Lane 1: GFP(-21) positive control; 2: molecular weight marker for SDS-PAGE; 3: empty TRAP-cage; 4: input (TRAP-cage with GFP); 5 and 8: flow through; 6 and 9: wash; and 7 and 10: elution. (c) Native PAGE gels showing encapsulation of GFP(-21) by unmodified TRAP-cage or TRAP-cage externally modified with Alexa-647 and PTD4. Lane 1: TRAP-cage with GFP(-21); 2: TRAP-cage with GFP(-21) decorated with Alexa-647; 3: TRAP-cage with GFP(-21) decorated with Alexa-647 and PTD4; and 4: molecular weight marker for native PAGE. Gels were stained for protein (upper panel) and analyzed by fluorescence detection of GFP (middle panel, exct. 488 nm) and Alexa-647 (bottom panel, exct. 647). Gels were imaged using a Biorad Chemidoc detector. (d) Negative stain TEM of TRAP-cage with GFP(-21) (left panel); TRAP-cage with GFP(-21) decorated with Alexa-647 (middle panel); and TRAP-cage with GFP(-21) decorated with Alexa-647 and PTD4 (right panel).

To fill TRAP-cage, we took advantage of the fact that the only significant patch of positive charge on the surface of the TRAP ring lies on the face lining the interior of the cage (Figure 1b,c). In principle, this could allow capture of negatively charged cargos via electrostatic interactions as has been demonstrated for other protein cages.^32−36^ The fact that the constituent TRAP rings do not assemble into TRAP-cage until the addition of gold(I)^8^ means that protein cargos below approximately 4 nm (the size of the fourfold holes in TRAP-cage^8^) have two possible routes for encapsulation—they may bind to TRAP rings prior to assembly or they may be added after TRAP-cage formation and allowed to diffuse into the cage through the fourfold holes. We chose negatively supercharged GFP(-21) as a model cargo (Figure 1d). This cylindrically shaped protein has a diameter of approximately 2.4 nm and is therefore expected to be able to diffuse into the assembled TRAP-cage (Figure 1e). His-tagged GFP(-21) was mixed with TRAP-cages and incubated overnight, followed by size exclusion chromatography purification for removal of remaining free GFP(-21). It was found that the two proteins associated as shown by co-migration of fluorescence signals on native gels (Figure 2a). To verify whether His-tagged GFP(-21) is inside the TRAP-cage and not bound to its exterior, we conducted a pull-down assay using Ni-NTA affinity chromatography, followed by western blot analysis. The observation that the GFP(-21) associated with TRAP-cage did not bind to the Ni-NTA column suggested successful encapsulation, making the His-tag inaccessible. This was further supported by a pull-down assay, which showed that the associated GFP(-21) was only available to interact with a Ni-NTA column after the cage was dissociated by the addition of a reducing agent (Figure 2b). These results strongly suggest encapsulation of GFP(-21) in TRAP-cage in either full or partial modes (partial encapsulation being the case where the GFP(-21) “plugs” the holes in TRAP-cage with the His-tags pointing to the interior). The number of GFP(-21) per cage was approximately 0.3 (Figure S1), similar to but lower than the range found in a number of other filled protein cages,^36−38^ although some have shown considerably greater numbers of cargos.^39^ Where the magnitude of charge complementarity is lower, a low cargo loading is expected, and while we have used a supercharged GFP, the cage interior bears only small patches of positive charges associated with the wild-type protein, likely accounting for the relatively low loading efficiency, which could, in future work, be addressed by modifying TRAP-cage further, such that it carries a higher density of positive charges within the cage interior. Alternatively, different methods of cargo capture (such as covalent attachment) could be explored, as described for other protein cages.^39,40^

### Decoration of TRAP-Cage with a Fluorescent Dye and with Cell-Penetrating Peptides

In order to be able to track TRAP-cage independently from its cargo, we labeled it with the Alexa-647 fluorescent dye. For this, we crosslinked the maleimide group on the dye with the 24 available cysteines lining the six 4 nm holes of TRAP-cage that are not involved in ring–ring interactions. By titration, we established the optimal amount of Alexa-647 to be added, this being the concentration at which the TRAP-cage is readily labeled and no free dye is present in the sample. It was assessed by native PAGE combined with fluorescent measurements to detect both GFP(-21) and Alexa-647 (Figure S3a). The yield of TRAP-cage labeling with Alexa-647 was quantified using fluorescence detection of labeled TRAP-cage of known concentration. The average number of Alexa-647 molecules bound to TRAP-cage was estimated to be 2 (Figure S3b). Although the cargo GFP(-21) contains three cysteine residues, control reactions showed no detectable labeling of GFP(-21) with Alexa-647 (Figure S3c).

We aimed to modify the TRAP-cage in order to promote its cell entry. For this, we chose PTD4 (YARAAARQARA)—an optimized TAT-based cell-penetrating peptide that shows a significantly improved ability to penetrate cell membranes, being more amphipathic with a reduced number of arginines and increased α-helical content.^41^ A number of studies have shown that coating nanoparticles with PTD4 promotes cell penetration.^42,43^ We attached the PTD4 derivative Ac-YARAAARQARAG to the amino groups on surface-exposed lysines of TRAP-cage. Based on the known structure,^8^ there are three such surface-exposed lysines per monomer on TRAP-cage, potentially allowing 792 peptides to be attached per cage. Calculations based on peptide peak areas from HPLC chromatograms were used to determine the initial amount of peptide and remaining amount after conjugation with TRAP-cage, indicating that 230 ± 22 PTD4 peptides are attached to one TRAP-cage (Figure S4b).

In reactions optimized for Alexa-647 labeling, we observed an increase in the apparent molecular weight of TRAP-cage after reaction with PTD4 (Figure 2c), as visualized by native PAGE. Negative stain TEM confirmed that the modified TRAP-cages retained their characteristic shape and size, being 22 nm in diameter (Figure 2d).

### Stability of TRAP-Cage and Effect on Cell Viability

Before embarking on cell delivery tests, we first assessed whether TRAP-cage was structurally stable, that is, did not disassemble under cell culture conditions. Stability was checked at 37 °C and in a 5% CO_2_ atmosphere in Dulbecco’s modified eagle medium (DMEM) without or with fetal bovine serum (FBS) at various concentrations. The results showed that the TRAP-cage structure is stable in DMEM culture medium within 18 h of incubation at 37 °C and 5% CO_2_ (Figure S5a).

In order to determine the effect of TRAP-cage on cell viability, alamarBlue assays were carried out. This test is based on the natural ability of viable cells to convert resazurin, a blue and nonfluorescent compound, into resofurin, a red and fluorescent molecule by mitochondrial and other reducing enzymes.^44^ Human cancer cell lines MCF-7 and HeLa were incubated in the presence of TRAP-cage and TRAP-cage filled with GFP(-21) and decorated with Alexa-647 and PTD4 peptide. The number of cells, TRAP-cage dose, and stimulation time used in cell viability tests correspond to the conditions under which the internalization of the TRAP-cage experiments was performed. Untreated cells were used as a control. The data showed that both unmodified TRAP-cage and TRAP-cage filled with GFP(-21) and decorated with Alexa-647 and PTD4 do not significantly affect the viability of MCF-7 and HeLa cells for at least 4 h of incubation (Figure S5b).

### Delivery of the Protein Cargo to Cells

**Figure 3:**
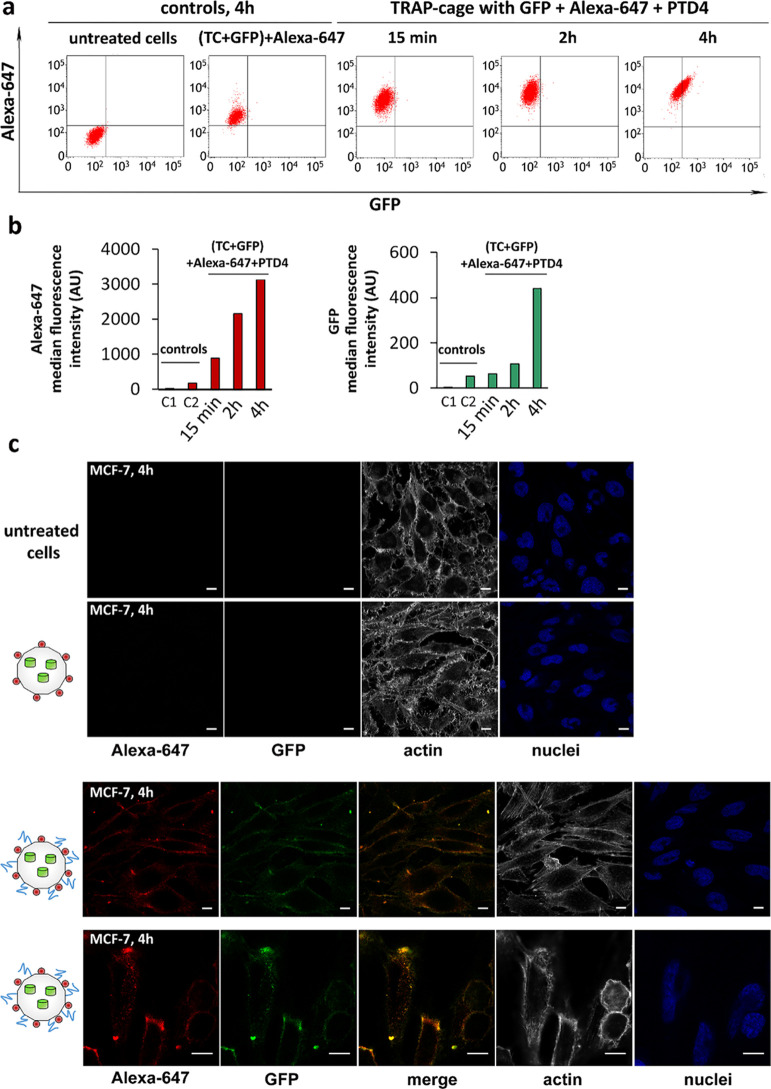
Delivery of TRAP-cage carrying GFP(-21) to MCF-7 cells. (a) Representative flow cytometry dot plots of MCF-7 cells after 4 h of treatment with Alexa-647-labeled TRAP-cage carrying GFP(-21) [denoted as (TC + GFP) + Alexa-647] and TRAP-cage with GFP(-21) labeled with Alexa-647 and PTD4 peptide [denoted as (TC + GFP) + Alexa-647 + PTD4] for 15 min, 2, and 4 h. The *x*-axis and the *y*-axis show the fluorescent intensities of GFP and Alexa-647, respectively. Untreated cells were used as the negative control. (b) Median fluorescence intensity of Alexa-647 and GFP positive cells treated with TRAP-cage carrying GFP and decorated with Alexa-647 or decorated with both Alexa-647 and PTD4 after 15 min, 2, and 4 h of incubations. Data are normalized to untreated cells and based on three independent experiments. Controls: C1: untreated cells and C2: cells incubated with (TC + GFP) + Alexa-647. (c) Confocal microscopy images of controls: untreated cells (first row), cells after 4 h of treatment with TRAP-cage filled with GFP(-21) labeled with Alexa-647 only (second row), TRAP-cage filled with GFP(-21) and labeled with Alexa-647 and PTD4 (third row) with additional single optical sections through the middle of the cell (fourth row). Actin filaments were stained with phalloidin conjugated to Alexa-568, and nuclei were stained with DAPI. Green channel: GFP; red channel: Alexa-647; blue channel: DAPI; and gray channel: Alexa-568. Merge: overlay image of red and green channels. Confocal images were taken at 63×; scale bar: 10 μm.

Delivery of TRAP-cage to cells was studied using human cancer cell lines MCF-7 and HeLa. Cells were incubated for different time periods with the purified TRAP-cages containing encapsulated GFP(-21) and labeled with Alexa-647 only or with Alexa-647 and PTD4 and analyzed by flow cytometry. The fluorescent signal due to both Alexa-647 and GFP(-21) increased with prolonged incubation time in both cell lines treated with TRAP-cage with GFP(-21) labeled with Alexa-647 and PTD4 peptide (Figure 3a,b). These results show that external modification of TRAP-cages with cell-penetrating peptides promotes their cell entry and effective cargo delivery. Interestingly, this effect was more pronounced in the case of the MCF-7 cell line compared to the HeLa cell line (Figure S6a,b).

In order to discriminate between fluorescent signals from TRAP-cages which were internalized in the cells and those which were adsorbed externally on the cell membrane, confocal microscopy was used. TRAP-cage containing GFP(-21) and labeled with Alexa-647 but lacking PTD4 was not observed in the cells. In contrast, TRAP-cage containing GFP(-21) and decorated with PTD4 showed a clear signal in the cell interior at 4 h after stimulation (Figures 3c and S6c).

The demonstration of delivery of the active protein cargo to cell interiors via an artificial protein cage is of interest given the small number of previous studies on artificial protein cage-mediated delivery of cargos to cells demonstrated for nonprotein cargos. Notably, Edwardson and co-workers showed that an artificial protein cage loaded with siRNA can be taken up by different mammalian cells and can release its cargo to induce RNAi and knockdown of target gene expression.^17^ In this case, the high gene-silencing efficiency together with low toxic effects indicated that a protein cage carrier has potential as a therapeutic delivery system. Encapsulation of protein cargos within artificial protein cages has previously been demonstrated by Votteler and colleagues.^21^ However, these cages were not shown to be able to directly deliver their cargo to cells. Instead, multiple copies of the cages themselves were used as cargos within lipid envelopes made in cells and purified as enveloped protein nanocages (EPNs), where the lipid envelope was derived from the host cell membrane. The EPNs were able to deliver the cages, meaning that entry to cells was achieved by the enveloping, host-derived membrane, not the protein cage.

### Intracellular Dynamics of TRAP-Cage

**Figure 4:**
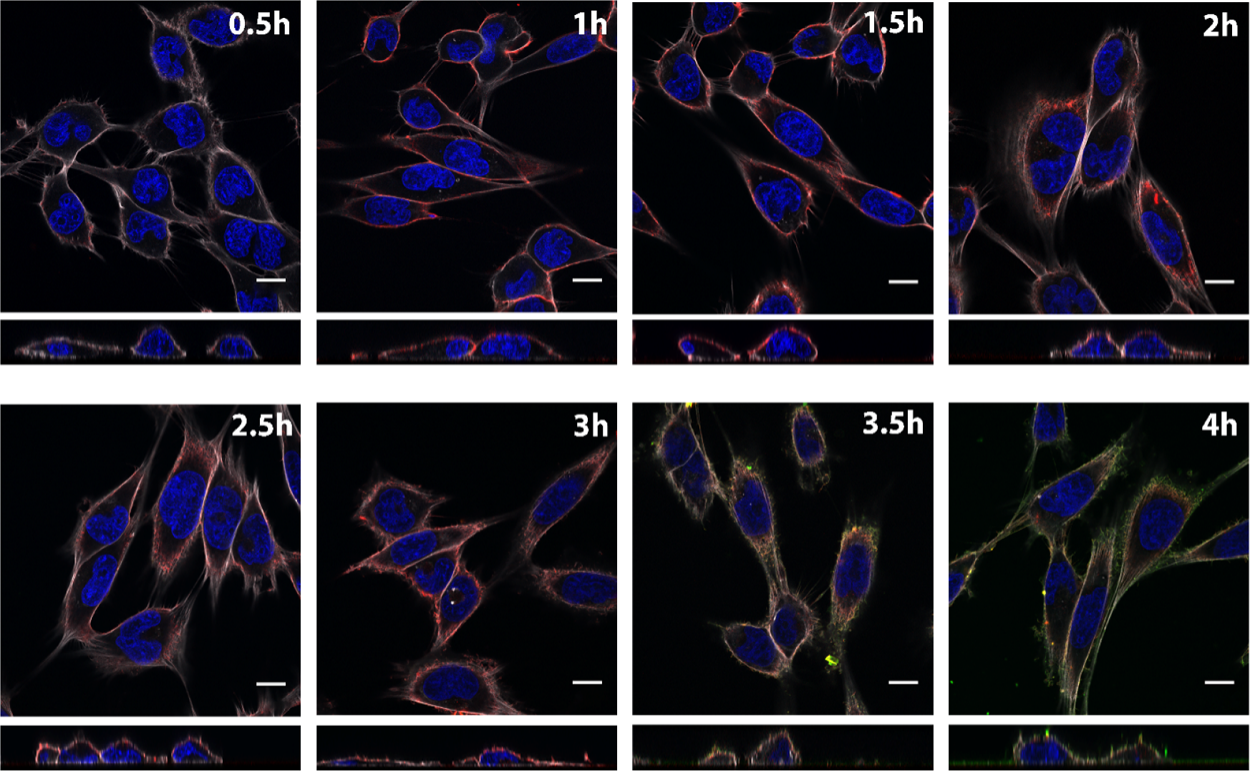
Tracking TRAP-cage and GFP(-21) in MCF-7 cells. Confocal microscopy merged images of cells incubated with TRAP-cage carrying GFP(-21) decorated with Alexa-647 and PTD4 and fixed at different time points. Actin was stained with phalloidin conjugated to Alexa-568, whereas DAPI was used for nuclear staining. Rectangular images beneath each main image are representative orthogonal views in the *yz* axis. Confocal images were taken at 63×; scale bar: 10 μm.

The high stability of TRAP-cage coupled with its ability to break apart in the presence of modest concentrations of cellular reducing agents suggests that TRAP-cage in the cytoplasm should readily disassemble, releasing the GFP(-21) cargo. As TRAP-cage and GFP(-21) possess discrete and trackable signals, we hypothesized that cage disassembly and release of GFP(-21) may be strongly inferred if the Alexa-647 and GFP(-21) signals became non-colocalized after cell entry. To assess this possibility, we tracked both signals over time after addition to MCF-7 and HeLa cancer cells. Notably, in both cell lines tested, during the first 90 min of incubation, TRAP-cage was mainly present at the cell boundaries, as indicated by the strong localization of the Alexa-647 signal there, and the GFP(-21) signal was barely detectable. However, after 3 h of incubation, the TRAP-cage signal (Alexa-647) became weaker and appeared to be distributed more evenly in the cell, whereas the GFP(-21) signal was clearly detectable, likely due to its release from the TRAP-cages (Figures 4 and S7). While differentiating between intracellular GFP(-21) in the cytoplasm and the endosome is challenging, evidence supporting cytoplasmic localization comes from the lag observed in the appearance of the GFP(-21) signal. IF GFP(-21) was localized in the endosome, we would expect degradation to result in a decrease rather than an increase in the signal over time.

To further confirm that TRAP-cage with GFP(-21) is successfully delivered to the cell interior and the cargo is released, we performed in-cell ELISA, where we detect GFP(-21) with a specific antibody, at several time points. These results demonstrated that the longer the cells were incubated with TRAP-cage filled with GFP(-21), the more detectable the GFP(-21) signal, suggesting that the cargo protein becomes more accessible for the antibody, that is, released or exposed by cage opening (Figure S11).

The change in relative signal strengths of TRAP-cage-associated Alexa-647 versus GFP(-21) once in the cell is suggestive of intracellular break-up of the cage and release of the cargo. A possible explanation is that when Alexa-647 and GFP(-21) are in close proximity to each other due to association with TRAP-cage, the GFP(-21) fluorescence may be decreased due to a (non-FRET) quenching effect from the dye. Once GFP(-21) is released by TRAP-cage disassembly, average GFP(-21) to Alexa-647 distances become larger, resulting in an increase in the detected GFP(-21) fluorescence. This possibility is supported by the observation that the signal from intracellular GFP(-21) is visibly brighter when it is delivered using TRAP-cage lacking Alexa-647 (Figure S6). Given the relatively low occupancy of GFP(-21) inside TRAP-cage and the fact that on average, each 24-ring cage bears only two Alexa-647 dye molecules, cage disassembly could, in principle, lead to separation of rings with electrostatically attached GFP(-21) from Alexa-647 simply by virtue of the fact that the ring to which GFP(-21) attached is not labeled with Alexa-647. For future potential use, this is not necessarily problematic, depending on the identity of the protein cargo, which will be active as long as any binding/active site is accessible.

## Conclusions

In this work, we demonstrated that an artificial protein cage can be used to deliberately encapsulate a protein cargo and deliver it to cell interiors. Importantly, the protein cages employed either in an unmodified form or externally decorated showed no significant effects on cell viability.

To achieve protein cage-encapsulated protein delivery to cells, we used our previously developed TRAP-cage^8^ having positively charged patches on its interior, to capture negatively supercharged GFP electrostatically through diffusion into the cage. Attempts to deliver filled cages to cells showed no evidence of penetration of TRAP-cages into cells if they were undecorated. In contrast, attachment of the cell-penetrating peptide PTD4 to the exterior of TRAP-cages resulted in significant penetration into cell interiors.

Overall, the work presented herein offers the first demonstration of protein delivery to cells mediated with a prototype system employing artificial protein cages (which we differentiate from engineered cages by virtue of the fact that artificial cages are protein cages whose constituent proteins do not naturally form a cage). An engineered cage, by contrast, is an existing cage (natural or artificial) that has been engineered, that is, altered to give it certain properties. We demonstrated a relatively low cargo-filling efficiency, and in the future study, this could be addressed by modifying TRAP-cage further such that it carries a higher density of positive charge within the cage interior. Alternatively, different methods of cargo capture (such as covalent attachment) could be explored, as described for other protein cages.^39,40^ Additionally, we anticipate further modification of TRAP-cage both to increase targeting specificity and to extend the range and usefulness of the encapsulated cargo. Finally, future studies will be required to pinpoint and track both the precise intracellular location of TRAP-cages and their quaternary state.

## Supporting Information Available

Estimating the number of His-tagged GFP(-21) molecules in the TRAP-cage; incubation in high salt concentration; TRAP-cage labeling with Alexa-647; PTD4 peptide synthesis and quantification; cell culture and cytotoxicity assessment of TRAP-cage; delivery of TRAP-cage to HeLa cells; influence of Alexa-647 on GFP(-21) fluorescence; in-cell ELISA (PDF)

## Abbreviations

TRAP: trp RNA-binding attenuation protein
GFP: green fluorescent protein
PTD4: protein transduction domain
CPP: cell-penetrating peptide
SDS-PAGE: sodium dodecyl sulfate-polyacrylamide gel electrophoresis
TEM: transmission electron microscopy
DMEM: Dulbecco’s modified Eagle medium
FBS: fetal bovine serum
