# Supplementary material for: Artificial Protein Cage Delivers Active Protein Cargos to the Cell Interior
Source: Biomacromolecules. 2021 Sep 9;22(10):4146–54. doi: 10.1021/acs.biomac.1c00630 (PMC8512669; doi:10.1021/acs.biomac.1c00630)
Supplement: Supplementary file 1 — bm1c00630_si_001.pdf [file bm1c00630_si_001.pdf]

SUPPORTING INFORMATION

An artificial protein cage with triggerable disassembly delivers active protein cargoes to cell interior

Antonina Naskalska[1], Kinga Borzęcka-Solarz[1]*, Jan Różycki[1], Izabela Stupka[1,2$],*

Michał Bochenek[1], Elżbieta Pyza[3], Jonathan G. Heddle[1#]

[1]Malopolska Centre of Biotechnology, Jagiellonian University, Krakow, Poland,

[2]Postgraduate School of Molecular Medicine, Żwirki i Wigury 61, 02-091 Warsaw, Poland

[3]Institute of Zoology and Biomedical Research, Jagiellonian University, Krakow, Poland.

[$]Current address: nCage Therapeutics, ul. Profesora Michała Bobrzyńskiego 14, 30-348, Krakow, Poland

*These authors equally contributed to this work

[#]email: jonathan.heddle@uj.edu.pl

Estimating the number of His-tagged GFP(-21) molecules in the TRAP-cage

Two methods were used for estimating the loading of GFP(-21):

1. Based on detection of GFP fluorescence in TRAP-cage filled with cargo. A GFP(-21) standard curve was prepared in the concentration range of 0-100 nM. The fluorescence spectra were acquired at 26 °C using a RF-6000 Shimadzu® Spectro Fluorophotometer with a fixed excitation wavelength at 488 nm and emission wavelength range of 495–550 nm, with an interval of 1.0 nm for λ_{em}, scan speed 6000 nm min^{-1}, λ_{ex} bandwidth 5 nm and λ_{em} bandwidth 5 nm. The fluorescence at emission maximum λ_{em} 510 nm was used for calculation. TRAP protein concentration was determined from absorbance at 280 nm to be 0.2 mg/mL (90 nM), in buffer containing 50 mM Tris, pH 7.9 and 150 mM NaCl. A TRAP-cage: GFP(-21) stoichiometry of 1: 0.28±0.07 was obtained (**Figure S1a**).

2. Densitometry analysis. Briefly, a series of His-tagged GFP(-21) dilutions (0.4 ng; 0.8 ng; 4 ng; 8 ng as measured by Nanodrop at wavelength 280 nm, which corresponds to 15; 30; 150; 300 fmoles) and TRAP-cage filled with cargo, sample (0.2 µg as measured by Nanodrop at wavelength 280 nm, which corresponds to 90 fmoles) were separated by SDS-PAGE and subjected to Western blotting (**Figure S1b**). The signal from His-tagged GFP(-21) protein was detected with anti-GFP antibody and secondary HRP-conjugated antibody in a chemiluminescence detector (Chemidoc, BioRad). Densitometry analysis using ImageLab (BioRad) software of the resulting blot showed that 0.6 ng (23 fmoles) of His-tagged GFP(-21) was present in 0.2 µg of TRAP-cage filled with cargo. The densitometry analysis yielded a TRAP-cage: GFP(-21) stoichiometry of approx. 1 : 0.25.

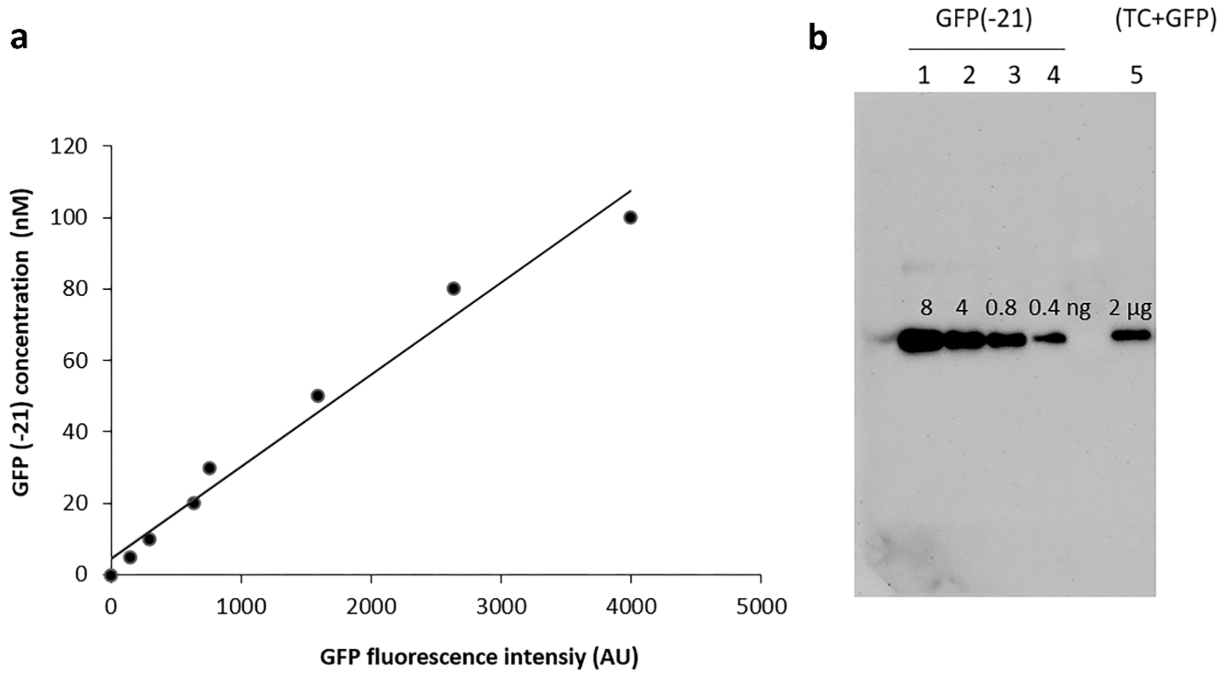

Figure S1. Estimating the number of His-tagged GFP(-21) molecules in TRAP-cage. (a) Standard curve obtained from fluorescence measurements of GFP(-21) protein, with the concentration range from 0 - 100 nM. Fitted with equation: $y = 0.0258x + 4.4$; $R^2 = 0.9786$. (b) Western blot used for band densitometry analysis. Lanes 1-4: GFP(-21); lane 5: TRAP-cage loaded with GFP(-21) (denoted as (TC+GFP)).

Incubation in high salt concentration

Purified TRAP-cage filled with GFP(-21) was buffer exchanged to 50 mM Tris, 500 mM NaCl pH 7.9 using an Amicon Ultra 10 kDa MWCO centrifugal filter unit (Millipore) and left at room temperature overnight. The next day buffer exchanged sample together with GFP(-21) and not buffer exchanged control samples were analyzed by native PAGE followed by Instant Blue gel staining and fluorescence detection (excitation at 488 nm, Chemidoc, BioRad) **(Figure S2).**

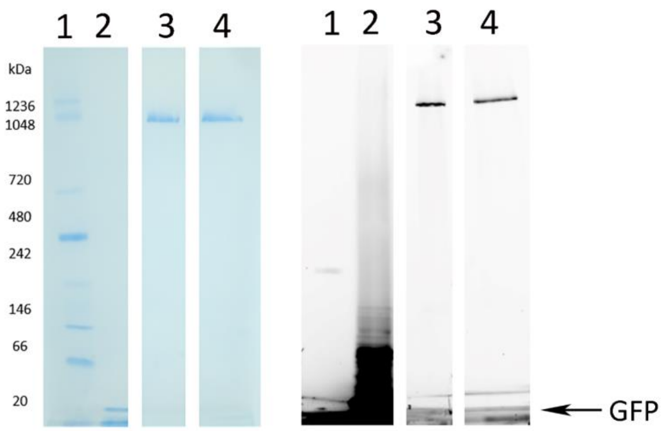

Figure S2. Partial leakage of GFP(-21) from TRAP cage after long term exposure to high salt concentrations. TRAP-cage-GFP(-21) was incubated overnight in buffer containing 500 mM salt and then separated in native PAGE. Gels were imaged using a Biorad Chemidoc for fluorescence detection at 488 nm (right side) and then stained with Coomassie blue (left side). Black arrow indicates the GFP(-21) band appearing after incubation in buffer containing 500 mM NaCl which may suggest weakening of the electrostatic interactions between the molecules leading to partial leakage of the GFP(-21) from the inside of the TRAP-cage. Lanes: 1: molecular weight marker; 2:control GFP (-21); 3- TRAP-cage with GFP(-21) incubated with 150 mM NaCl; 4- TRAP-cage with GFP(-21) incubated with 500 mM NaCl.

Optimization of TRAP-cage labeling with Alexa-647

The optimal interaction ratio of maleimide-conjugated Alexa-647 to TRAP-cage was assessed by titration (**Figure S3a**). Briefly, aliquots of TRAP-cage loaded with GFP(-21) (11.36 nM) were mixed with maleimide-conjugated Alexa-647 ranging from 0.1 µM to 100 µM. Samples were then separated by native gel electrophoresis and visualized by fluorescence detection in a Chemidoc, with excitation at 647 nm. Reactions where no free Alexa-647 is present in the sample and no GFP

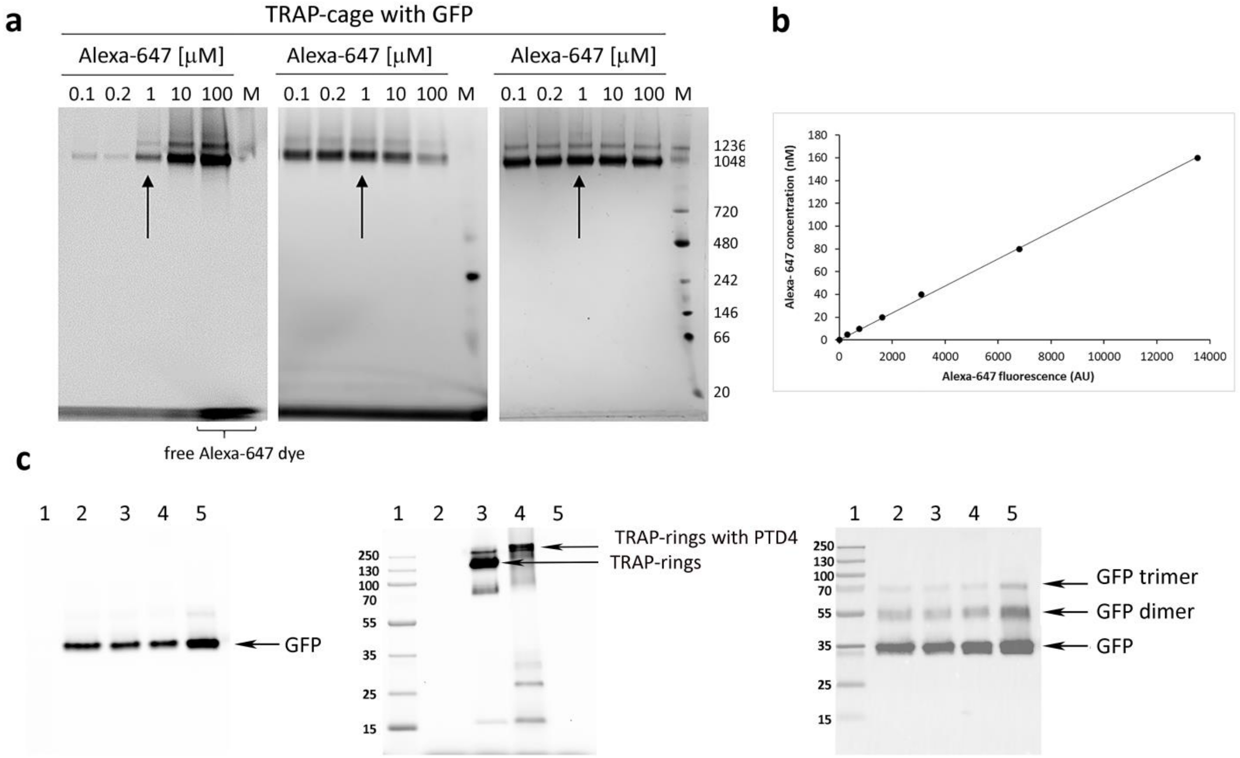

Figure S3. TRAP-cage with GFP(-21) labeling with Alexa-647 (a) Native PAGE gels showing TRAP-cage carrying GFP(-21) subject to optimization of the Alexa-647 conjugation reaction. Gels were analysed by fluorescence detection of Alexa-647 (left panel, exct. 647) and GFP(-21) (middle panel, exct. 488 nm) and stained for proteins (right panel). Arrows show optimal decoration conditions used in further experiments. TRAP-cage runs at approximately the same position as the 1048 KDa marker. Fainter band at approximately 1236 kDa band is likely to be a larger TRAP aggregate or assembly. (b) Standard curve used to calculate the amount of Alexa-647 conjugated to TRAP cage. (c) SDS-PAGE gel comparing TRAP-cages carrying GFP(-21) either with no decoration, decorated with Alexa-647 or decorated with both Alexa-647 and PTD4. Left: detection at 488 nm; middle: detection at 647 nm; right: Western blot of the same samples detected with anti-GFP antibody. Lanes: 1: molecular weight marker for SDS-PAGE electrophoresis; 2: TRAP-cage with GFP(-21); 3: TRAP-cage with GFP(-21) decorated with Alexa-647; 4: TRAP-cage with GFP(-21) decorated with Alexa-647 and PTD4; 5: GFP(-21) - positive control. Gels and Western blot were imaged using Biorad Chemidoc instrument.

interference with the Alexa-647 signal is observed, were considered as optimal decoration conditions and used in further experiments. The yield of TRAP-cage labeling with Alexa-647 was quantified using fluorescence detection of labeled TRAP protein of known concentration.

All fluorescence spectra were obtained using a Shimadzu RF6000 Spectro Fluorophotometer with LabSolutions RF software. Relative quantum yields were determined using the following parameters: excitation at 650 nm, emission spectrum was recorded between 660 and

700 nm, scan speed: 6000 nm min−1, data interval 1nm, excitation bandwidth: 5.0 nm, emission

$C_{TRAP\ Cage}$ [nM]	$F_{Alexsa\ 650/665}$ [AU]	C_{Alexa} [nM]	Alexa : TRAP Cage
**90**	16122	190	2.1 : 1
**75**	13413	158	2.1 : 1
**70**	11448	135	1.9 : 1

Table 1. Calculations of number of Alexa-647 molecules conjugated to TRAP-cage.

band width: 5.0 nm. The relative fluorescence at 665 nm was used to calculate the total amount of Alexa-647 dye attached to TRAP-cage based on a calibration curve obtained for free dye at the concentration range 0 - 160 nM (**Figure S3b**). Based on the analysis of three different samples, average labeling yield of 2 Alexa-647 molecules per one molecule of TRAP cage was calculated (**Table S1**).

Additionally, to rule out a possibility of direct GFP labeling by Alexa-647, TRAP-cage loaded with GFP(-21) with and without Alexa-647 labelling were subjected to denaturing gel separation and Western blotting followed by detection with anti-GFP antibody. No band shift from potential interaction of GFP with Alexa-647 dye was observed (**Figure S3c**).

PTD4 peptide synthesis and quantification

PTD4 peptide derivative (Ac-YARAAARQARAG, for simplicity called PTD4 in the text) was synthesized at 0.1 mmol scale using a Liberty Blue automated microwaved synthesizer (CEM, USA), according to the Fmoc-based solid phase peptide synthesis methodology. Fmoc-Gly-Wang resin (100-200 mesh, substitution 0.70 mmol/g, Novabiochem, Germany) was swelled overnight with dichloromethane (DCM)/dimethylformamide (DMF) (1:1). Fmoc-deprotection was performed with 25% morpholine in DMF for 5 min at 85 °C. Coupling reactions were performed as per manufacturer’s recommended protocol using DIC/oxyma activators with a fivefold excess

of Fmoc-protected amino acid derivatives for 5 min at 85 °C. Double coupling was applied for all Fmoc-Arg (Pbf) coupling. N-terminal acetylation was performed on resin with 10% acetic anhydride in DMF at 60 °C. Cleavage from the resin and side chain deprotection were achieved by treatment with TFA/Triisopropylsilane (TIS)//water (94:3:3) for 4 h with vigorous shaking at 30 °C . The resin was filtrated and TFA was evaporated under a mild nitrogen stream. The crude peptide was precipitated by addition of cold diethyl ether, followed by centrifugation (3000 rpm, 10 min). The residue was washed with cold ether (2x) and ethyl acetate (2x). Precipitated crude peptide was dried *in vacuo* overnight. Crude peptide was dissolved in 8 M urea and purified on an Agilent 1260 RP-HPLC using semi-preparative C18 (10x150 mm) column (Cosmosil, Nacalai tesque). Collected peptide-containing fractions were lyophilized. Purified peptide was analyzed on an analytical C18 column (Zorbax SB-C18 5mm 4.6x150 mm, Agilent) in a linear gradient of 0 – 20% of acetonitrile with 0.1% TFA for 30 min at flow rate 1.0 ml/min. Peak signals were detected at 220 and 280 nm.

To determine the yield of cage decoration with peptide we used two independent approaches.

1. We took advantage of the observed shift of TRAP-cage band on native PAGE after peptide conjugation due to attached peptides. Based on the masses of molecular markers, a standard curve (log MW vs RF factor) was determined. Since protein migration under native conditions is dependent not only on molecular mass but also on protein pI, running buffer pH, and type and percentage of gel matrix, the apparent TRAP cage mass (actual MW 2.2 kD) was around 1500 kDa. For the calculation we included a correction factor that is the ratio of real cage mass over the value calculated based on the standard curve. Typically, this correction factor was around 1.5. For the calculation, estimated molecular masses of cage before and after conjugation were multiplied by the correction factor and the mass

difference was divided by peptide molecular weight (1.3kD) resulting the number of peptides attached to the cage. Calculations based on the three different native gels gave average estimation of 247+/-17 peptides per cage (**Figure S4a**).

2. The second approach was based on RF C18 HPLC analysis. The samples (before and after conjugation) of a known initial amount of peptide and TRAP were injected and analysed by HPLC. Integrated peptide peak areas were used to determine the initial amounts and amounts remaining after conjugation, allowing total amount of peptide attached to TRAP-cage to be calculated. The integration of peak areas of three injections detected at three wavelengths (210, 230 and 280 nm) gave results of 230+/-22 peptide molecules per TRAP cage (**Figure S4b and Table S2**).

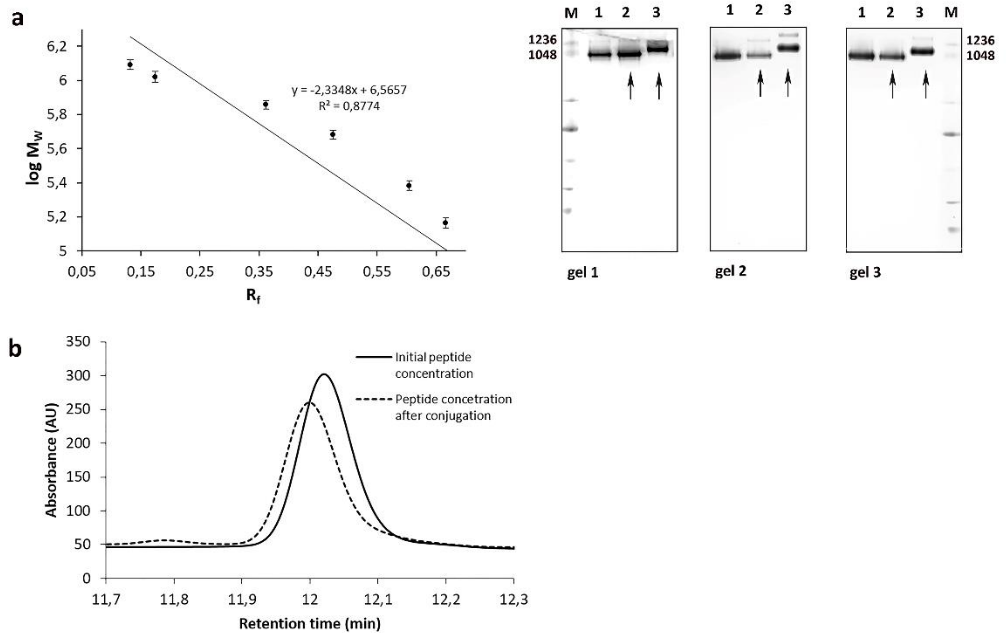

Figure S4. Quantification of PTD4 peptide conjugated with TRAP-cage filled with GFP(-21). (a) Analysis of differences in undecorated and decorated TRAP-cage migration in gels based on masses of molecular markers. Left hand side, standard curve (log MW vs RF factor) used for calculations, right hand side, native gels showing TRAP-cage before (lane: 1, 2) and after decoration with PTD4 (lane: 3), M - molecular weight marker for native PAGE. Arrows show analysed bands (b) Representative HPLC chromatogram of AcPTD4 peptide used for determination of number of peptide conjugated to TRAP-cage. Solid line represents peak of initial peptide concentration; dashed line refers to peptide peak after conjugation to TRAP-cage.

Peptide	A_{210}	A_{230}	A_{280}	Retention time [min]
AcPTD4 before conjugation	1427	541	86	12.0
AcPTD4 after conjugation	1220	462	74	11.9
Peptide per TRAP cage	220	207	223	-

Peptide	A_{210}	A_{230}	A_{280}	Retention time [min]
AcPTD4 before conjugation	1576	570	91	12.0
AcPTD4 after conjugation	1297	485	77	12.0
Peptide per TRAP cage	269	225	232	-

Final average: 230 +/- 22

Supplementary Table 2. Calculations of number of PTD4 peptides conjugated to TRAP-cage

Cell culture and cytotoxicity assessment of TRAP-cage

HeLa and MCF-7 cells were cultured in Dulbecco's Modified Eagle Medium (DMEM, Sigma) supplemented with 10% FBS (EURx), 100 µg/ml streptomycin, 100 IU/ml penicillin (Gibco). The culture was maintained at 37 °C under 5% CO_2.

To test TRAP-cage stability in the culture medium, purified sample was added to DMEM medium containing 0, 2 and 10% fetal bovine serum (FBS) and incubated at 37 °C under 5% CO_2 for 2 h, 6 h and 18 h. Samples were subsequently analyzed by native PAGE followed by Instant blue gel staining (**Figure S5a**).

Cell viability after TRAP-cage treatment was determined using the alamarBlue test (VWR). Cells were cultured in 96-well plates at a density of 2.5×10^4 cells per well. Next, cells were treated with 5 µg (0.6 nM) TRAP-cage, TRAP-cage filled with GFP(-21) and decorated with Alexa-647 and PTD4 in 50 mM HEPES with 150 mM NaCl pH 7.5 supplemented with 10% FBS for 4 h. After the treatment, the buffer with TRAP-cage was removed and 10 µl of alamarBlue diluted in 90 µl DMEM medium was added per well, and cells were incubated for the next 3 h at 37 °C under 5% CO_2. Resazurin, the active component of alamarBlue, was reduced to the highly fluorescent

compound resorufin only in viable cells and absorbance (excitation 570 nm, emission 630 nm) of this dye was recorded. Nontreated cells were used as a negative control (**Figure S 5b**). All samples were measured in triplicate, in three independent experiments.

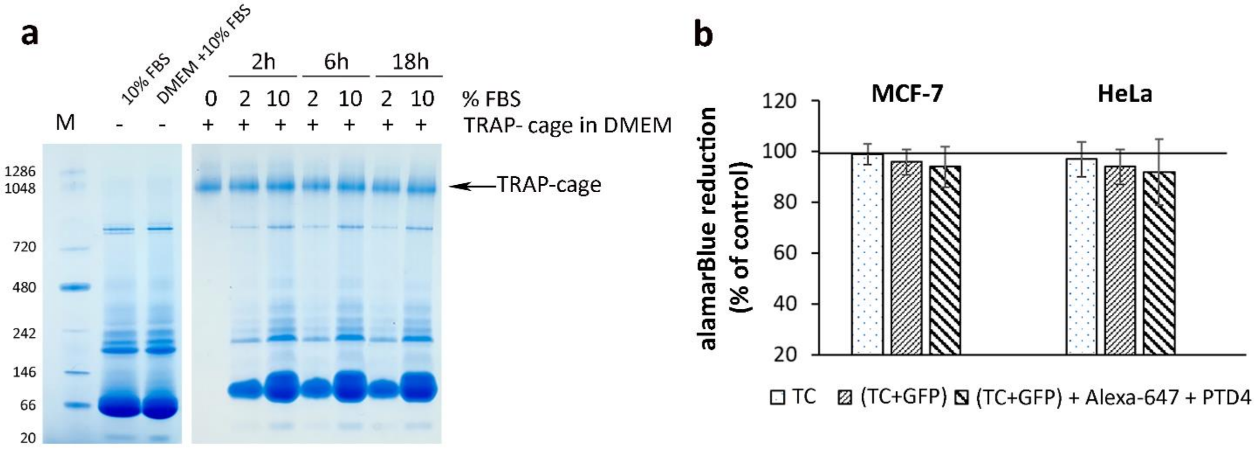

Figure S5. TRAP-cage stability in culture medium and cell viability test. (a) Native PAGE gels showing controls: 10% FBS and DMEM culture medium with 10% FBS (left gel) and TRAP-cage stability in DMEM culture medium without and with 2% and 10% FBS during 18 h incubation (right gel). Gels were imaged using Biorad Chemidoc instrument. (b) Cell viability of MCF-7 and HeLa cells after 4 h exposure to empty TRAP-cage, TRAP-cage loaded with GFP(-21) and TRAP-cage with GFP(-21) decorated with Alexa-647 and PTD4. Results from three independent experiments, measured in triplicate. M = molecular weight marker for native electrophoresis; TC: empty TRAP-cage; (TC+GFP): TRAP-cage filled with GFP(-21); (TC+GFP) + Alexa-647 + PTD4: TRAP-cage with GFP(-21) and decorated with Alexa-647 and PTD4.

Delivery of TRAP-cage to HeLa cells

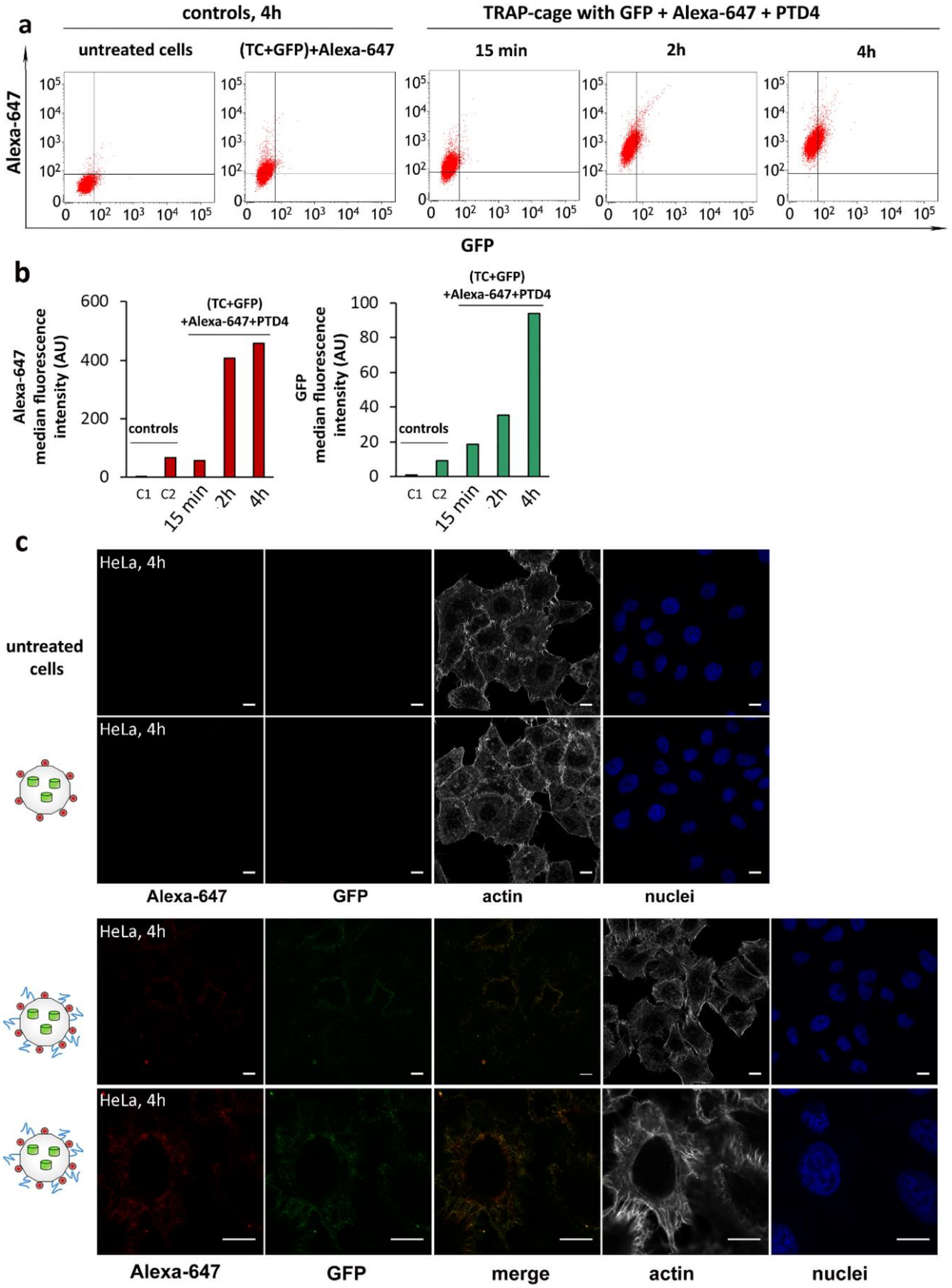

Figure S6 (Previous page) Delivery of TRAP-cage with GFP(-21) to HeLa cells. (a) Representative flow cytometry dot plots of HeLa cells after treatment with Alexa-647 labeled TRAP-cage with GFP(-21) for 4 h (denoted as (TC+GFP) + Alexa-647) and Alexa-647 labeled TRAP-cage with GFP(-21) and PTD4 (denoted as (TC+GFP) + Alexa-647 + PTD4) for 15 min, 2 h and 4 h. The x-axis and the y-axis show the fluorescence intensities of GFP(-21) and Alexa-647 respectively. Untreated cells were used as the negative control. (b) Median fluorescence intensity of Alexa-647 and GFP(-21) positive cells treated with (TC+GFP) + Alexa-647 and (TC+GFP) + Alexa-647 + PTD4 after 15 min, 2 h and 4 h incubation. Data are normalized to untreated cells and based on three independent experiments. Controls: C1, untreated cells and C2, cells incubated for 4 h with (TC+GFP) + Alexa-647. (c) Confocal microscopy images of controls: untreated cells (first row), cells after 4 h treatment with TRAP-cage filled with GFP(-21) labelled with Alexa-647 only (second row) and cells after 4 h incubation with TRAP-cage filled with GFP(-21) and labelled with Alexa-647 and PTD4 (third row) with additional single optical sections through the middle of this cell (fourth row). Confocal images were taken at 63x objective, scale bar: 10 µm. Actin filaments were stained with phalloidin conjugated to Alexa-568 and nuclei were stained with DAPI. Green channel – GFP(-21); red channel – Alexa-647; blue channel – DAPI; grey channel – Alexa-568. Merge – shows overlay image of red and green channels.

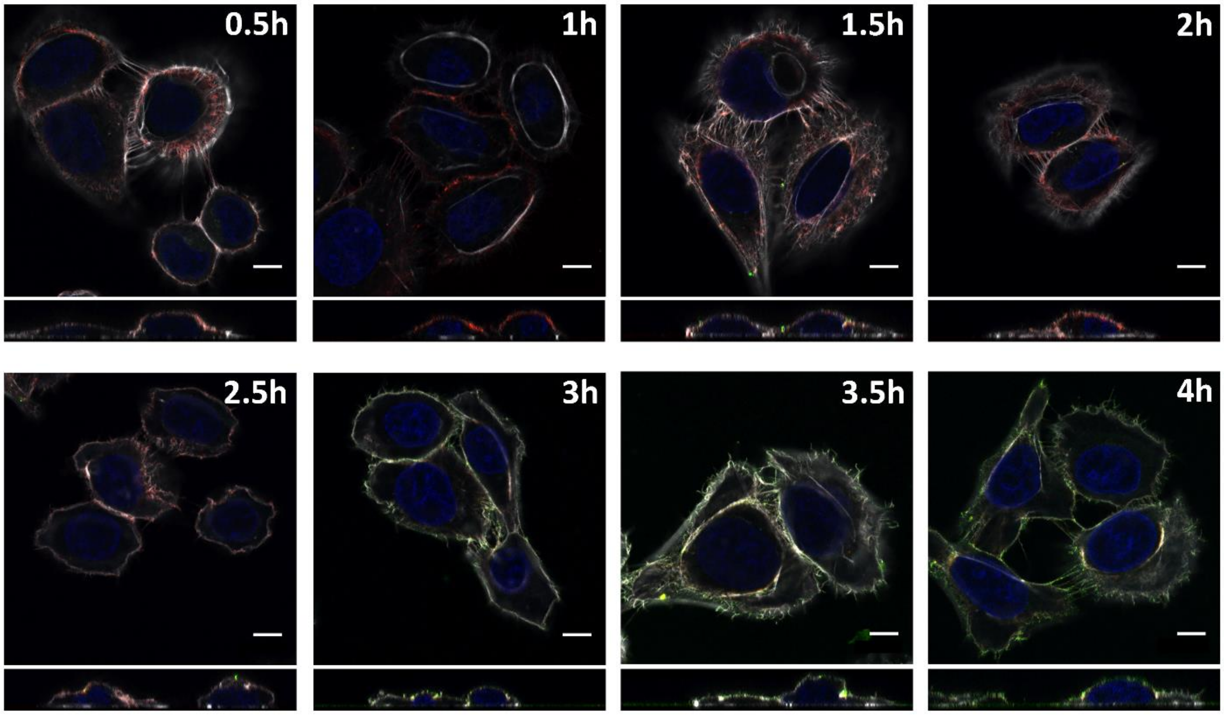

Figure S7. Tracking TRAP-cage and GFP(-21) in HeLa cells. Confocal microscopy images of merged single optical sections of cells incubated with TRAP-cage with GFP(-21) labeled with Alexa-647 and PTD4 and fixed at different time points. Actin was stained with phalloidin conjugated to Alexa-568 whereas DAPI was used for nuclear staining. Rectangular images are representative orthogonal views in the yz axis. Confocal images were taken at 63x objective, scale bar: 10 μm.

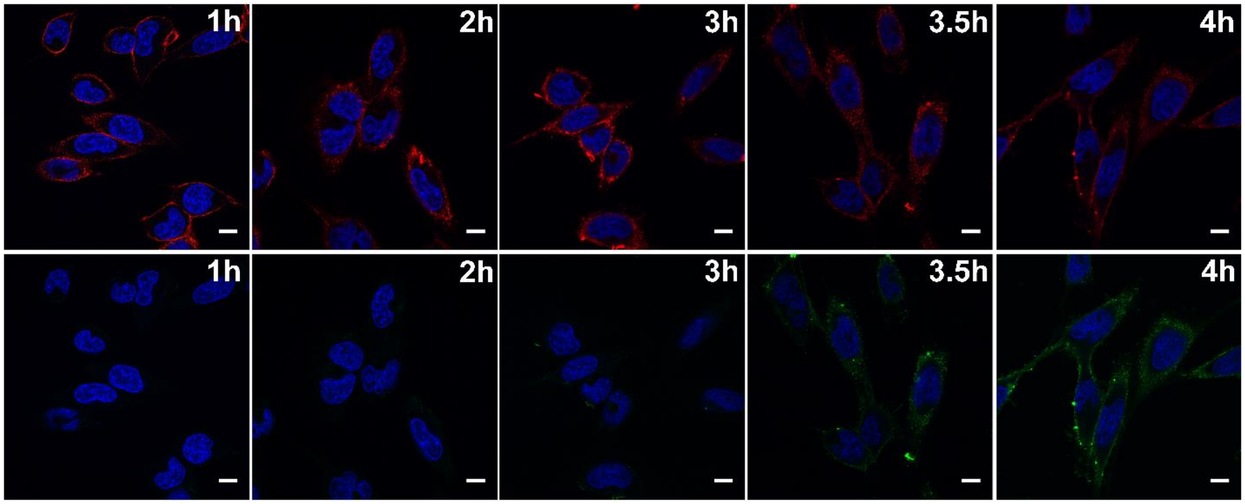

Figure S8. Tracking TRAP-cage and GFP(-21) in MCF-7 cells. Confocal microscopy images of cells incubated with TRAP-cage carrying GFP(-21) decorated with Alexa-647 and PTD4 and fixed at different time points. Results are presented in individual red (upper panel) and green (bottom panel) channels from single optical sections through the middle of the cell. DAPI was used for nuclear staining. Confocal images were taken at 63x, scale bar: 10 μm.

* This is an additional representation of the results shown in Figure 4 in the main manuscript.

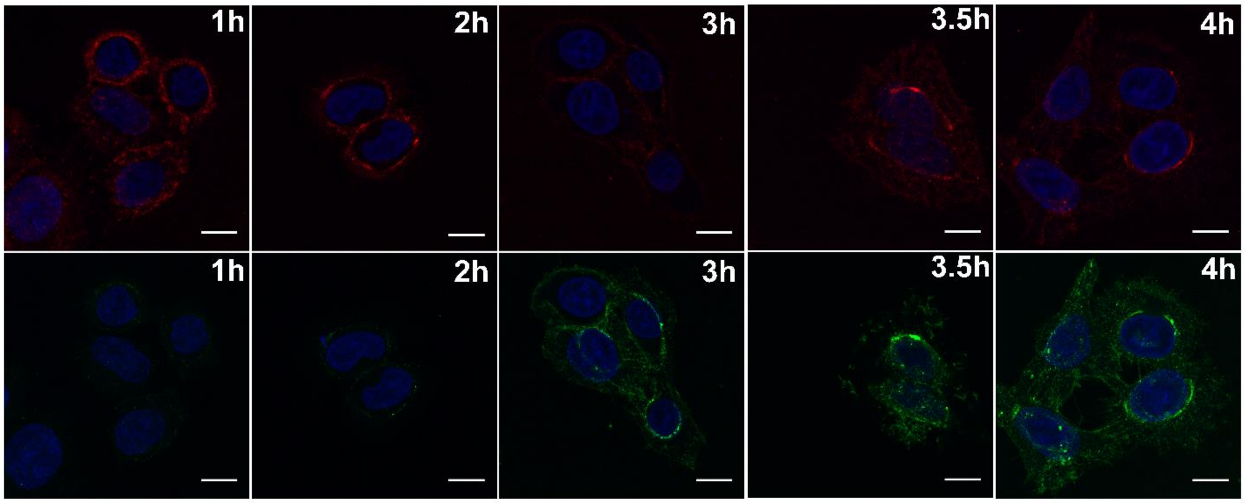

Figure S9. Tracking TRAP-cage and GFP(-21) in HeLa. Confocal microscopy images of cells incubated with TRAP-cage carrying GFP(-21) decorated with Alexa-647 and PTD4 and fixed at different time points. Results are presented in individual red (upper panel) and green (bottom panel) channels with maximum intensity projection from stack images, separated 0.67 μm. DAPI was used for nuclear staining. Confocal images were taken at 63x objective, scale bar: 10 μm.

Influence of Alexa-647 of GFP(-21) fluorescence

To assess the potential influence of Alexa-647 on GFP(-21) fluorescence (suggested by **Figure S2a**, middle panel) we compared, by confocal microscope imaging, TRAP-cages filled with cargo where the cages compared were either decorated with PTD4 peptide only or were fully decorated (PTD4 and Alexa-647) (**Figure S10a**). Briefly, cells were treated with the respective samples as described in Materials and Methods. Next, cells were fixed and stained following the protocol described above. The fluorescence intensity in the green channel was quantified with ImageJ. Calculations of the mean fluorescence intensity (**Figure S10b**) took into account the background signal from each field of view.

Additionally, in-solution fluorescence of GFP(-21) encapsulated in the fully decorated TRAP-cage was compared to the fluorescence of the cargo in the TRAP-cage without Alexa-647

using a RF-6000 Shimadzu Spectro Fluorophotometer. As shown in **Figure S10c** presence of the Alexa-647 dye on the TRAP-cage results in approximately 30% reduction in the fluorescence of its cargo.

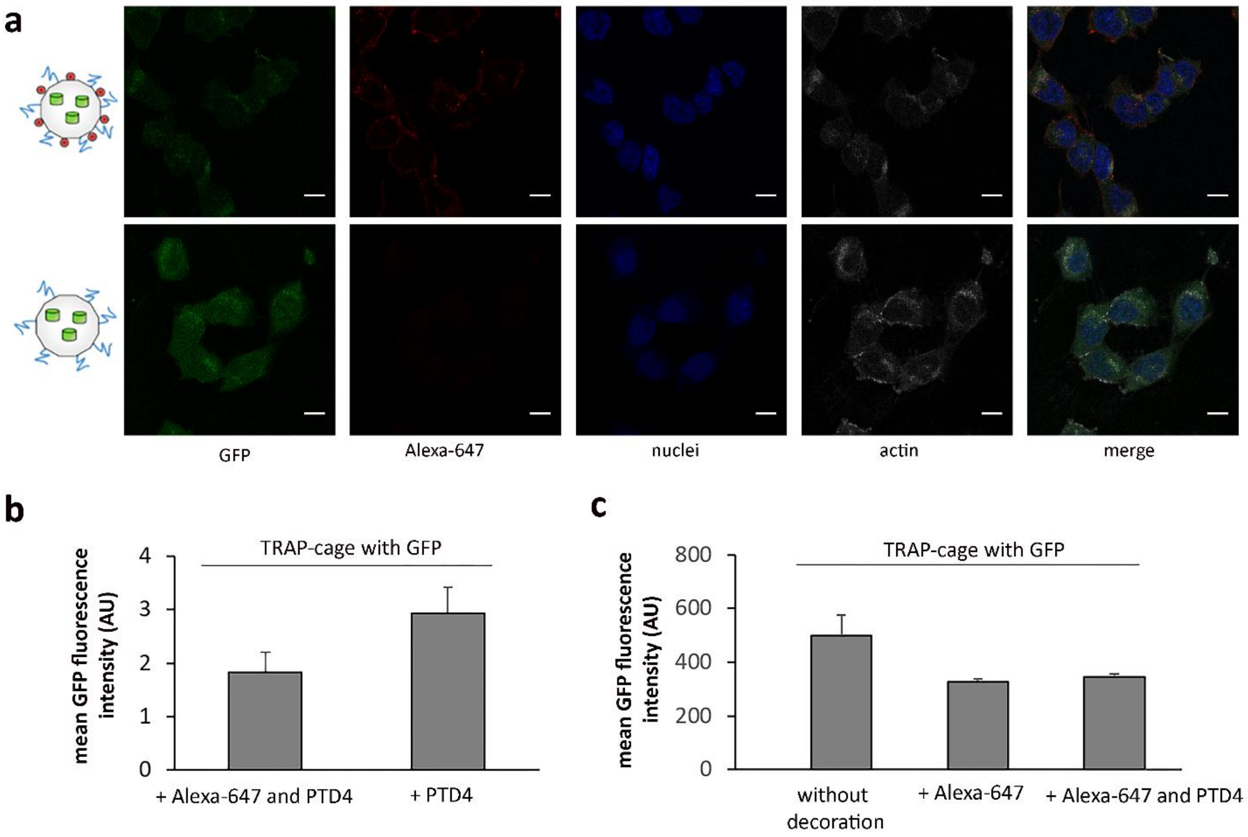

Figure S10. Influence of Alexa-647 of GFP(-21) fluorescence. (a) Cells were exposed to (TC+GFP) labeled with Alexa-647 and PTD4 (upper row) or (TC+GFP) labeled with PTD4 only (lower row). Actin filaments were stained with phalloidin conjugated to Alexa-568 and nuclei were stained with DAPI. Green channel – GFP(-21); red channel – Alexa-647; blue channel – DAPI; grey channel – Alexa-568; (scale bar: 10 μm). (b) Mean GFP(-21) fluorescence intensity registered from three different fields of view for samples where cells were exposed to (TC+GFP) labeled with Alexa-647 and PTD4 or (TC+GFP) labeled with PTD4 only. The fluorescence intensity was quantified with ImageJ, considering background intensity subtraction. (c) Mean fluorescence of GFP(-21) encapsulated in the undecorated and fully decorated TRAP cage, measured in solution.

In cell ELISA

To provide evidence that the cage opens in cells we performed in cell-ELISA with anti-TRAP and anti-GFP antibody detection. We made the reasonable assumption that cargo GFP is not accessible to the large antibody unless the cage is opened. Once the cage is open, it would be expected that the signal from the secondary antibody would increase in time. For this, $3x10^4$ cells (both MCF-7 and HeLa) were seeded into wells of 96-wells plate. TRAP-cage filled with GFP(-21) and decorated with PTD4 peptides was added to cells at several time points (0.5h, 1h, 2h, 3h, 4h of incubation). Then cells were washed thrice with PBS (0.01% Tween-20), fixed with 4% paraformaldehyde solution (15 min, at room temperature) and permeabilized with 0.5% Triton-X100 in PBS (5 min, at room temperature). Wells were blocked with 5% rat serum (overnight in 4 °C) and proteins were detected with mouse anti-GFP antibody (St. Johns's Laboratory; 1: 2000; 1.5h) or anti-TRAP rabbit polyclonal serum (Eurogentec, 1:2500; 1.5h), followed by anti-mouse HRP conjugated (Jackson's; 1: 4000; 1h) and anti-rabbit HRP conjugated (Cell Signaling; 1:10 000, 1h) secondary antibodies. Colorimetric reaction was developed by adding 3,3',5,5'-tetramethylbenzidine substrate, stopped by 0.1M HCl and registered in plate reader (Tecan) at wavelength 450 nm.

A clear increase of signal from GFP(-21) detection over time was registered (**Figure S11**). Interestingly, a similar effect was observed with anti-TRAP antibody. Importantly this anti-TRAP antibody has been raised by immunizing animals with TRAP protein, (not the whole cage) and therefore is more reactive with disassembled then intact cage. These results are indicative of TRAP-cage opening to make GFP(-21) accessible once inside cages.

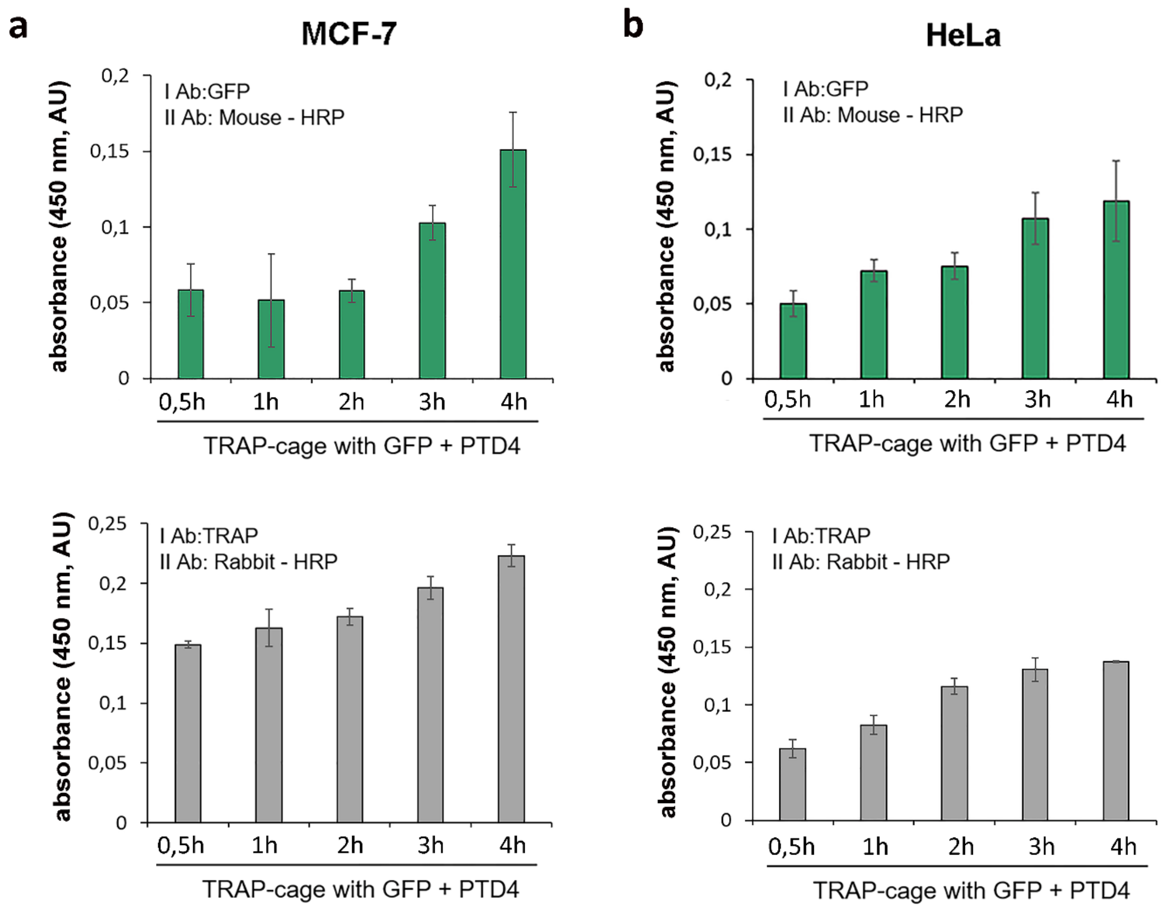

Figure S11. In cell ELISA: (a) MCF-7 and (b) HeLa cells were incubated with TRAP-cage with GFP(-21) decorated with PTD4 peptides for several time periods (ranging from 0.5h to 4h). After washes, cells were fixed and the TRAP-cage and the GFP(-21) were detected using specific antibodies. Data are normalized to untreated cells. Each time point was tested in triplicate.
